# Liberation or Extraction of Impacted Teeth

**Published:** 1905-04-15

**Authors:** M. H. Cryer


### LIBERATION OR EXTRACTION OF IMPACTED TEETH.

BY M. H. CRYER, M.D. D.D.S.

The positions into which the developing permanent teeth are forced, because of abnormal conditions of the deciduous teeth, account for many mal-positions assumed by the permanent teeth. The cone-shaped teeth are very liable to get wrong directions. Some are deeply set, others assume quite superficial relations. Failure in the absorption of deciduous roots or any physiologic function is enough to create mal-position. Pathologic conditions are also important factors. Sometimes supernumerary teeth or odontocees may produce impaction of the permanent teeth. Malocclusion is, however, the great factor in shortening the arches and producing impacted teeth.

In my experience the teeth are liable to impaction in the following order: Lower third molar, upper cuspid, upper third molar, upper central incisor, lower second bicuspid, upper second bicuspid, lower cuspid.

The third molar germ, before the second molar crown has completely formed, is located in almost a horizontal position in the rannes of the lower jaw, and as the jaw develops it may be carried forward in that position, so that when it develops its occlusal surface comes squarely forward against the distal side of the second molar crown. In some cases it is tipped down and really develops upside down.

The cuspid tooth germ is located well up and deeply in the jaw, and is the last of the twelve teeth to assume its place in the arch. If there should be premature loss of the deciduous cuspid, or extensive inflammation producing considerable cicatricial tissue and dense process, it is not strange that the cuspid should be diverted, especially in view of the shape and size of the forming crown. In many cases these teeth have been found in the palate, and often completely without the normal line of occlusion.

The extraction of impacted teeth is usually indicated and is always more or less difficult and sometimes hazardous. The X-ray pictures are of the greatest value in locating missing teeth as well as in determining exact positions. If the form and location are exactly known, the operative procedure is more simple. It will be found usually that the bone about impacted teeth is hard and adheres closely to the teeth, which makes it difficult to dislodge the tooth, even when exactly located. The overlying bone can be cut away with suitable burs, and thus facilitate extraction without fracture in many cases. Where the lower third molars have come forward in a horizontal position, their cusps lock into the distal of the lower second molar so that it is necessary to use a disk and cut away the cusps entirely before trying to lift out the tooth which will only wedge tighter without this operation. These impacted teeth very often have deformed or greatly accentuated roots, which add greatly to the difficulties of extracting them. The upper third molars are especially liable to varieties of unexpected root formations. The lower third molars are sometimes retarded in eruption or forced into impacted conditions by malocclusion of the teeth. When the lower teeth are in distal relation to the upper, there is no inducement for the jaw to grow forward and so make room for the third molar. In fact, the jaw either stands still in its development or actually retrogrades because of the forces of occlusion and the labial and buccal muscle pressure. In extracting impacted third molars the elevator is the most useful instrument aided when necessary by the engine to remove overlying hard structures or to free the tooth from contact with adjoining teeth.—Extract from *Journal of American Medical Association.*

